# Predictors of target initial teicoplanin trough concentration attainment in patients with hematological malignancies: a retrospective cohort study

**DOI:** 10.1186/s40780-026-00550-w

**Published:** 2026-02-05

**Authors:** Kazuki Deguchi, Norihiro Sakurai, Toya Matsui, Mayuko Itoya, Makoto Miyoshi, Hiroshi Kawaguchi, Yasutaka Nakamura

**Affiliations:** 1 Department of Pharmacy, Osaka Metropolitan University Hospital, 1-5-7 Asahi-machi, Abeno-ku, Osaka, Japan; 2 Department of Infection Control Science, Graduate School of Medicine, Osaka Metropolitan University, 1-4-3 Asahi-machi, Abeno-ku, Osaka, Japan

**Keywords:** Teicoplanin, Hematological malignancy, Therapeutic drug monitoring

## Abstract

**Background:**

Teicoplanin (TEIC) is frequently used at Osaka Metropolitan University Hospital to treat infections caused by gram-positive cocci in patients with hematological malignancies. However, in certain cases, the initial trough concentrations failed to reach the target level, despite appropriate loading doses. Therefore, this study aimed to identify the factors associated with achieving target concentrations based on the initial trough level following TEIC loading in patients with hematological malignancies.

**Methods:**

We retrospectively analyzed patients with hematological malignancies treated with TEIC between January 2016 and December 2022. Patients were classified into a target trough attainment group (trough ≥ 15 µg/mL) and a non-attainment group (trough < 15 µg/mL) according to the initial trough concentration measured on day 4 after administering the loading doses. Demographics, clinical laboratory data, TEIC dosing, and concomitant medications were compared between groups. Factors independently associated with the target trough concentration were assessed using multivariate logistic regression analysis.

**Results:**

Among the 176 patients, 90 (51%) achieved the target trough concentration, while 86 (49%) did not. Multivariate analysis identified febrile neutropenia, history of bone marrow transplantation within 30 days, presence or absence of tacrolimus (TAC) coadministration, and cumulative TEIC dose over the first 3 days as independent predictors of target attainment. The target concentration attainment rate was only 22% among patients with febrile neutropenia who received a TEIC cumulative loading dose of < 40 mg/kg over the first 3 days after initiation and did not receive TAC coadministration.

**Conclusions:**

Achieving adequate TEIC concentrations early in therapy is critical for the successful treatment of immunocompromised patients with hematological malignancies. Our findings indicated that the likelihood of target trough concentration attainment markedly reduced in patients with multiple risk factors for subtherapeutic exposure. Accordingly, higher doses should be administered, particularly to patients with these factors.

## Background

Teicoplanin (TEIC), a glycopeptide antibiotic with antimicrobial activity against methicillin-resistant Staphylococcus aureus and methicillin-resistant coagulase-negative staphylococci (MRCNS), is widely used to treat infections such as sepsis, pneumonia, and skin and soft tissue infections [1–3]. It has a high plasma protein-binding rate of approximately 90% and a long half-life of 30–180 h [1, 2]. Therefore, loading doses are required at the start of therapy to rapidly achieve therapeutic concentrations [3–5]. According to Japanese guidelines, the initial dose regimen is determined based on body weight and renal function. Doses are administered every 12 h on the first and second days of treatment, and then switched to once daily administration from the third day to implement loading doses. The measurement of trough concentrations and therapeutic drug monitoring (TDM) are recommended prior to TEIC administration on the fourth day after treatment initiation [4]. The pharmacokinetic/pharmacodynamic parameters correlated with the clinical efficacy of TEIC are defined as the area under the concentration–time curve (AUC)/minimum inhibitory concentration (MIC) [6]. However, because evaluating the AUC in clinical practice is challenging, the trough concentration is used as a surrogate marker. In recent years, a target trough concentration of 15–30 µg/mL has been recommended for TEIC, and in severe cases and complex infections (such as endocarditis and osteoarticular infections), a concentration of ≥ 20 µg/mL is recommended [4, 7–11].

Patients with hematological malignancies are at high risk of infection owing to the underlying disease and the immunosuppressive effects of multiple intensive cytotoxic chemotherapies [12–14]. Many patients with hematological malignancies undergo bone marrow transplantation (BMT) and receive multiple immunosuppressive, antifungal, antiviral, or other concomitant medications during their treatment course, which may affect renal function and contribute to the variability in the pharmacokinetic parameters of renally excreted drugs. Therefore, in patients with hematological malignancies who develop infections such as febrile neutropenia (FN), bloodstream infections, or pneumonia suspected to be caused by resistant gram-positive bacteria, treatment with TEIC is often preferred over vancomycin owing to its lower risk of renal dysfunction [15–17]. Nevertheless, predicting the pharmacokinetics of TEIC in patients with hematological malignancies is considered difficult due to disease-related pathophysiological changes [3, 18] and the effects of concomitant medications. Accordingly, even with appropriate loading doses, the target blood concentration is occasionally not achieved early in clinical practice. In patients with hematological malignancy, initiating treatment with the optimal TEIC loading dose and achieving effective trough concentrations early is not only a key factor in treatment success but also reduces the risk of developing resistance to glycopeptide antibiotics when concentrations remain below the effective trough concentration [19]. Previous studies have reported an association between TEIC trough concentration and efficacy. One study identified a cut-off value of 15.2 µg/mL for predicting treatment success in patients with hematological malignancy who develop FN [20], while Byrne et al. reported that a concentration of ≥ 20 µg/mL is necessary [3]. However, studies on the factors related to achieving the target TEIC trough concentration in patients with hematological malignancies remain limited. Factors associated with low TEIC trough concentrations include increased renal clearance, elevated distribution volume, FN, and acute myeloid leukemia (AML) [18, 21, 22]. However, the underlying mechanisms and the appropriate initial dosing regimens remain unclear.

Therefore, this study aimed to examine the background factors affecting blood concentrations after initial loading doses in patients with hematological malignancies and determine the optimal dosage for individualized treatment.

## Methods

### Study design

We conducted a single-center, retrospective, observational study of patients with hematological malignancies who received intravenous TEIC at the Osaka Metropolitan University Hospital between January 2016 and December 2022. The study focused on data from patients who received TEIC and had their initial trough concentrations measured at steady state (on day 4 after initial administration). The exclusion criteria were as follows: (1) initial trough concentration measured on a day other than day 4 after initiating administration; (2) initial trough concentration measured within 18 h of previous administration or unavailable trough concentration data; (3) estimated glomerular filtration rate (eGFR) < 60 mL/min/1.73 m^2^ or receiving renal replacement therapy at the start of TEIC administration; (4) renal dysfunction during the period from TEIC treatment initiation to the first trough concentration measurement, defined as serum creatinine of ≥ 0.3 mg/dL or a 1.5-fold increase from baseline following TEIC administration; (5) no accurate physical information within the last month; and (6) age < 18 years.

### Data collection and definitions

The following data were retrospectively obtained from electronic medical records: patient characteristics, including sex, age, weight, body surface area, body mass index (BMI), diagnosis, transplant history, and clinical laboratory data; TEIC administration information; and concomitant medications. In this study, the loading dose was calculated as the cumulative loading dose administered during the first 3 days of treatment. The dosing interval on days 1 and 2 was once or twice daily, and only once daily on Day 3. Clinical laboratory values were classified by grade using CTCAE Ver. 5.0. FN was defined as an axillary temperature of ≥ 37.5 °C, an absolute neutrophil count < 500 cells/µL, or a count < 1,000 cells/µL with a predicted decrease to < 500 cells/µL within 48 h.

### Factors affecting TEIC trough concentration

Based on the initial TEIC trough concentration, patients were classified into two groups: the target trough attainment group (≥ 15.0 µg/mL) and the non-attainment group (< 15.0 µg/mL), and their data were compared. Factors associated with achieving target trough concentrations were analyzed using multivariate logistic regression. This study incorporated variables that were clinically associated with the outcome and previously reported risk factors in the multivariable model. The cut-off value of 15 µg/mL was based on the Antimicrobial TDM Clinical Practice Guidelines and previously reported data [2].

Additionally, we performed subgroup analyses on patients who had multiple factors identified as significant in multivariate analysis and examined the proportion of patients reaching target trough concentrations among those with factors affecting multiple trough concentrations.

### Statistical analysis

Continuous and categorical variables were compared using the Mann–Whitney U, Fisher’s exact, or chi-squared tests. Logistic regression analysis was performed using the target attainment and non-attainment groups with the initial TEIC concentration as the dependent variable. Patient background, factors previously reported to be associated with low TEIC trough concentrations [3, 5, 6], and clinically significant factors were included as explanatory variables. Data analyses were performed using EZR (Saitama Medical Center, Jichi Medical University, Saitama, Japan), a graphical user interface for R (R Foundation for Statistical Computing, Vienna, Austria). This is a modified version of the R Commander, designed to add statistical functions that are frequently used in biostatistics. Two-tailed *P* < 0.05 were considered statistically significant.

## Results

### Patient characteristics

Table 1 summarizes the characteristics of patients in the target trough attainment and non-attainment groups. A total of 90 and 86 patients were assigned to the target trough attainment and non-attainment groups, respectively. The target trough attainment group had lower body weight, BMI, and percentage of FN than the non-attainment group. Notably, the target trough attainment group received a significantly higher cumulative loading dose. Upon comparing underlying diseases, the proportion of patients with myelodysplastic syndrome was high in the non-attainment group. The two groups did not differ significantly in terms of the clinical laboratory parameters (Table 2). Among the patients receiving concomitant medication in both groups, the proportion of patients receiving voriconazole (VRCZ) was high in the target trough attainment group (Table 3). No other patient characteristics or concomitant medications were significantly associated with the target trough attainment.

**Table 1:** Patient characteristics

Characteristic		Target trough non-attainment group (*n* = 86)	Target trough attainment group (*n* = 90)	*P*-value
Age	Median [IQR]	49 [34.8, 58.0]	50.5 [37.0, 58.0]	0.824
Male	n (%)	54 (62.8)	56 (62.2)	1.000
Weight (kg)	Median [IQR]	61.5 [54.4, 69.9]	57.6 [51.4, 66.1]	0.021
Cumulative loading dose (mg/kg)	Median [IQR]	36.9 [31.7, 41.3]	42.9 [38.4, 48.2]	< 0.001
Body mass index (kg/m^2^)	Median [IQR]	21.7 [19.9, 23.3]	19.2 [19.2, 23.0]	0.034
Febrile neutropenia	n (%)	68 (79.1)	56 (62.2)	0.022
Bone marrow transplantation within 30 days	n (%)	41 (47.7)	30 (33.3)	0.074
Underlying conditions				
Acute myeloid leukemia	n (%)	32 (37.2)	42 (46.7)	0.224
Myelodysplastic syndrome	n (%)	21 (24.4)	9 (10.0)	0.015
Acute lymphoblastic leukemia	n (%)	8 (9.3)	17 (18.9)	0.085
Adult T-cell lymphoma	n (%)	4 (4.7)	1 (1.1)	0.203
Diffuse large B-cell lymphoma	n (%)	3 (3.5)	2 (2.2)	0.677
Acute lymphoblastic lymphoma	n (%)	3 (3.5)	0 (0.0)	0.115
Aggressive NK-cell leukemia	n (%)	2 (2.3)	0 (0.0)	0.237
Blastoid plasma cell-like dendritic cell neoplasm	n (%)	2 (2.3)	0 (0.0)	0.237
Chronic myeloid leukemia	n (%)	2 (2.3)	4 (4.4)	0.683
Multiple myeloma	n (%)	2 (2.3)	0 (0.0)	0.237
Mixed-phenotype acute leukemia	n (%)	2 (2.3)	1 (1.1)	0.614
Hodgkin lymphoma	n (%)	1 (1.2)	2 (2.2)	1.000
Lymphocytic lymphoma	n (%)	1 (1.2)	0 (0.0)	0.489
Aplastic anemia	n (%)	0 (0.0)	5 (5.6)	0.059
Extranodal NK/T-cell lymphoma	n (%)	0 (0.0)	1 (1.1)	1.000
Hypoplastic leukemia	n (%)	0 (0.0)	1 (1.1)	1.000
Myelofibrosis	n (%)	0 (0.0)	1 (1.1)	1.000

**Table 2:** Clinical laboratory values

			Target trough non-attainment group (*n* = 86)	Target trough attainment group (*n* = 90)	*P*-value
Creatinine	Grade 0	n (%)	85 (98.8)	90 (100)	0.489
	Grade ≥ 1	n (%)	1 (1.2)	0 (0)	
eGFR (mL/min/1.73 m^2^)	≥ 90.0	n (%)	26 (30.2)	38 (42.2)	0.135
	< 90.0	n (%)	60 (69.8)	52 (57.8)	
Albumin	Grade < 3	n (%)	85 (98.8)	90 (100)	0.489
	Grade ≥ 3	n (%)	1 (1.2)	0 (0)	
AST	Grade < 3	n (%)	86 (100)	90 (100)	-
	Grade ≥ 3	n (%)	0 (0)	0 (0)	
ALT	Grade < 3	n (%)	79 (91.9)	87 (96.7)	0.204
	Grade ≥ 3	n (%)	7 (8.1)	3 (3.3)	
Total bilirubin	Grade < 3	n (%)	79 (91.9)	87 (96.7)	1.000
	Grade ≥ 3	n (%)	7 (8.1)	3 (3.3)	
Neutrophil	Grade < 3	n (%)	7 (8.1)	13 (14.4)	0.237
	Grade ≥ 3	n (%)	79 (91.9)	77 (85.6)	
Hemoglobin	Grade < 3	n (%)	57 (66.3)	60 (66.7)	1.000
	Grade ≥ 3	n (%)	29 (33.7)	30 (33.3)	
Platelets	Grade < 3	n (%)	11 (12.8)	19 (21.1)	0.164
	Grade ≥ 3	n (%)	75 (87.2)	71 (78.9)	

ALT, alanine aminotransferase; AST, aspartate aminotransferase; eGFR, estimated glomerular filtration rate

**Table 3:** Concomitant medication

Drug name		Target trough non-attainment group (*n* = 86)	Target trough attainment group (*n* = 90)	*P*-value
Immunosuppressant				
Tacrolimus	n (%)	24 (27.9)	31 (34.4)	0.440
Cyclosporine	n (%)	12 (14.0)	13 (14.4)	1.000
Mycophenolate mofetil	n (%)	8 (9.3)	13 (14.4)	0.413
Antibiotics				
4th -gen cephalosporin	n (%)	22 (25.6)	30 (33.3)	0.322
Carbapenem	n (%)	35 (40.7)	30 (33.3)	0.350
Fluoroquinolone	n (%)	4 (4.7)	6 (6.7)	0.747
TAZ/PIPC	n (%)	26 (30.2)	22 (24.4)	0.489
ST	n (%)	23 (26.7)	21 (23.3)	0.728
Antifungal drugs				
Voriconazole	n (%)	0 (0.0)	10 (11.1)	0.004
Itraconazole	n (%)	5 (5.8)	2 (2.2)	0.405
Fluconazole	n (%)	40 (46.5)	35 (38.9)	0.384
L-AMB	n (%)	1 (1.2)	0 (0.0)	0.982
Micafungin	n (%)	16 (18.6)	14 (15.6)	0.736
Caspofungin	n (%)	24 (27.9)	27 (30.0)	0.889
Posaconazole	n (%)	1 (1.2)	1 (1.1)	1.000
Antiviral drugs				
Acyclovir	n (%)	49 (57.0)	61 (67.8)	0.186
Ganciclovir	n (%)	0 (0.0)	3 (3.3)	0.260
Letermovir	n (%)	20 (23.3)	17 ( 18.9)	0.599
Hoscalnet	n (%)	2 (2.3)	1 (1.1)	0.968
Others				
NSAIDs	n (%)	1 (1.2)	1 (1.1)	1.000
ARB	n (%)	1 (1.2)	0 (0.0)	0.982

ARB, angiotensin II receptor blocker; L-AMB, liposomal amphotericin B; NSAIDs, non-steroidal anti-inflammatory drugs; ST, sulfamethoxazole trimethoprim; TAZ/PIPC, tazobactam/piperacillin

### Factors affecting TEIC trough concentration

Logistic regression analysis was performed using the target trough attainment group and the non-attainment group as dependent variables, and age, sex, FN, history of BMT within 30 days, concomitant use of tacrolimus (TAC), albumin (Alb), TEIC cumulative loading dose, and eGFR ≥ 90 mL/min/1.73 m^2^ as explanatory variables (Table 4). The factors included in the multivariate analysis were selected based on previous reports and comprised those affecting TEIC blood concentration and renal function [3, 7, 23, 24]. For the explanatory variables used in the multivariate analysis, we assessed multicollinearity beforehand using the Variance Inflation Factor (VIF) and confirmed that no significant multicollinearity existed between variables. While patient characteristics such as age and sex were not significant, FN and a history of BMT within 30 days were associated with low TEIC trough concentrations. Furthermore, concomitant TAC use and cumulative loading dose were associated with the attainment of the target TEIC concentration.

**Table 4:** Multivariate analysis: factors affecting the attainment of target trough levels

	Odds (95% CI)	*P*-value
Age	0.86 (0.25‒2.93)	0.807
Sex	1.01 (0.47‒2.17)	0.973
Febrile neutropenia	0.40 (0.17‒0.93)	0.034
BMT within 30 days	0.33 (0.14‒0.82)	0.017
Combination therapy with tacrolimus	3.33 (1.30‒8.53)	0.012
Serum albumin level	1.32 (0.64‒2.74)	0.456
Cumulative loading dose	1.20 (1.12‒1.27)	< 0.001
eGFR ≥ 90 mL/min/1.73 m^2^	0.53 (0.23‒1.18)	0.119

BMT, bone marrow transplantation; CI, confidence interval; eGFR, estimated glomerular filtration rate

### Patients with multiple factors associated with TEIC trough concentration

**Fig. 1:**
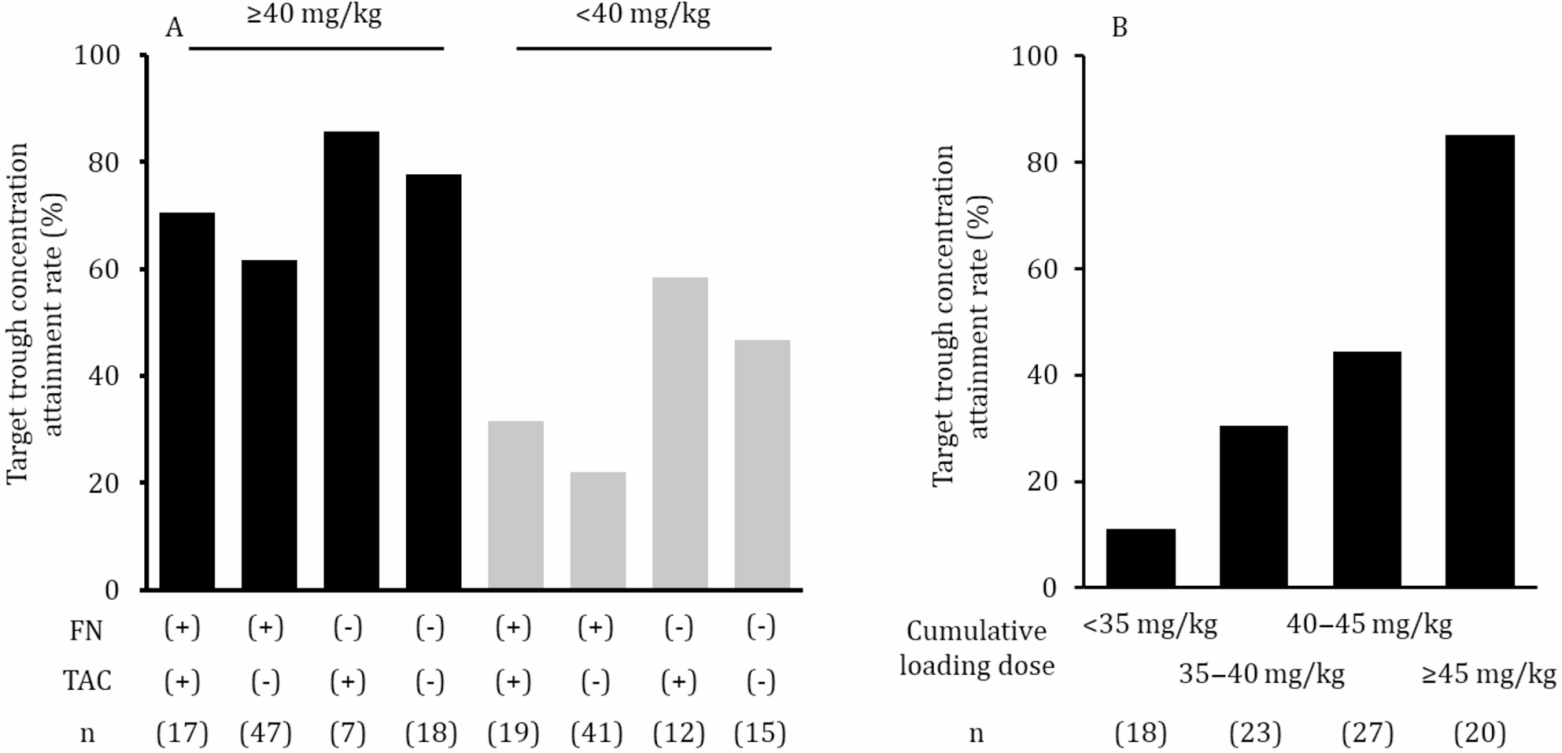
Target trough concentration attainment rate. (**A**) The combined effects of factors influencing trough concentrations. (**B**) The cumulative loading doses in patients with FN not taking TAC concomitantly. FN, febrile neutropenia; TAC, tacrolimus

Figure 1A illustrates the target concentration attainment rate for each patient with multiple explanatory variables that significantly affected blood concentration attainment based on the results of the multivariate logistic regression analysis. Patients who received a cumulative loading dose of < 40 mg/kg tended to have a lower likelihood of achieving the target trough concentration. Notably, the proportion of patients who achieved the target trough concentration was the lowest (22.0%, 9/41 cases) among those who developed FN and did not receive TAC concomitantly. Next, we extracted only patients who developed FN, had the lowest target concentration attainment rate, and did not receive TAC, and examined the target concentration attainment rate for each total load dose per body weight (Fig. 1B). The target concentration attainment rate increased in a dose-dependent manner with TEIC, reaching 85.0% at a cumulative loading dose of ≥ 45 mg/kg. On the other hand, no cases of renal impairment were observed at a cumulative loading dose of ≥ 45 mg/kg (data not shown).

## Discussion

In this study, we identified the factors predicting the attainment of an initial TEIC trough concentration of ≥ 15 µg/mL in patients with hematological malignancies. Independent predictors included a cumulative loading dose within the first 3 days of administration, TAC coadministration, FN, and a history of BMT within 30 days. Among patients with hematological malignancies who developed FN, those who did not receive TAC had the lowest target trough attainment rate (22.0%) when the cumulative loading dose was < 40 mg/kg. Therefore, we examined the cumulative loading dose needed to achieve the target trough concentration in patients with FN not receiving concomitant TAC and found that a cumulative loading dose of ≥ 45 mg/kg over three days yielded an attainment rate of ≥ 80%.

TEIC requires loading doses to achieve therapeutic blood levels early, and several loading dose regimens have been explored in recent years. For example, Ueda et al. have reported a regimen of 10–12 mg/kg administered at 12-h intervals or 35‒40 mg/kg administered 48 h after treatment initiation [21]. However, patients with hematological malignancies were not included in these reports, and predicting TEIC pharmacokinetics in this population is challenging owing to fluctuating blood concentrations caused by physiological changes and the effects of concomitant medications. Byrne et al. found that a TEIC trough concentration of 19.6 µg/mL was required in patients with hematological malignancies who developed catheter-related bacteremia caused by MRCNS [3]. Likewise, Sato et al. reported that a TEIC trough concentration of ≥ 15.2 µg/mL was necessary in patients with hematological malignancies who developed FN [20]. Although effective blood concentrations have been explored, studies examining predictors for achieving target blood concentrations in patients with hematological malignancies remain limited [3, 5, 25]. Therefore, in this study, we compared and examined factors, such as patient characteristics, underlying diseases, history of BMT, laboratory values, and concomitant medications.

Univariate analysis revealed significant differences in body weight, cumulative loading dose, BMI, FN, and VRCZ between groups. Next, multivariate analysis was performed to identify independent factors associated with achieving an initial TEIC trough concentration of ≥ 15 µg/mL. We incorporated variables considered clinically relevant to the outcomes and previously reported risk factors into the multivariate model regardless of their statistical significance in the univariate analysis. Therefore, factors examined in this study were patient background (sex and age), FN, history of BMT within 30 days, concomitant TAC use, Alb, cumulative loading dose, and renal function. Alb was included because TEIC has a high protein-binding rate, and its blood concentration reportedly correlates with serum Alb [25]. Additionally, TAC was included because it is frequently used concomitantly as an immunosuppressive agent during BMT and may potentially affect renal function. Based on the results of multivariate analysis, FN, history of BMT within 30 days, concomitant TAC use, and cumulative loading dose were identified as independent predictors for achieving an initial TEIC trough concentration of ≥ 15 µg/mL. Also, VRCZ, which was significant in the univariate analysis, was not included in the multivariate analysis. The reason for this exclusion is that VRCZ is primarily metabolized hepatically, and to our knowledge, there are no reports indicating a direct pharmacokinetic interaction between VRCZ and TIEC. Furthermore, although we considered the potential impact on renal function due to increased TAC trough concentrations with VRCZ and TAC co-administration, this combination was used in few patients, and no clinically significant changes in renal function were observed. Therefore, the observed association was likely attributable to patient background imbalances and limited sample size rather than true causality, and was not incorporated in the multivariate analysis.

FN and AML have been reported as risk factors for decreased blood TEIC concentration [18, 21, 22]. In FN and AML, systemic inflammatory responses mediated by inflammatory cytokines are thought to increase cardiac output and renal blood flow, thereby enhancing the renal clearance of drugs [5]. In addition, increased production of inflammatory cytokines elevates vascular permeability, which increases the distribution volume of TEIC, a water-soluble drug, making it difficult to achieve higher blood concentrations during loading doses. In this study, FN was identified as a negative factor for achieving a trough concentration of ≥ 15 µg/mL, consistent with previous reports. We also identified a history of BMT within the past 30 days and concomitant TAC use as independent risk factors. Patients who have undergone BMT are at a high risk of developing FN and are prone to increased distribution volume and renal clearance owing to increased vascular permeability caused by the release of inflammatory cytokines. Therefore, TEIC blood concentrations may not readily increase in patients during the early post-BMT period. In addition, TAC has been reported to temporarily reduce eGFR by constricting the afferent arterioles of the renal glomerulus via increased sensitivity of angiotensin II receptors [23, 26]. Therefore, TEIC blood concentrations may increase when administered concomitantly with TAC. On the other hand, in the present study, TAC coadministration was not significant in the univariate analysis but emerged as a significant factor in the multivariate analysis. This finding suggests that TAC coadministration should be interpreted not as a factor directly contributing to the attainment of target TEIC blood concentrations, but rather as an association identified after adjustment for confounding factors in the multivariate analysis. In this study, renal function (eGFR ≥ 90 mL/min/1.73 m²) was not an independent factor affecting TEIC blood concentration. This may be due to the exclusion of patients with renal impairment (eGFR < 60 mL/min/1.73 m^2^) and the inclusion of renal function as a nominal measure in the analysis. Furthermore, the reduction in eGFR associated with the concomitant TAC use was temporary and may not have significantly impacted renal function.

Additionally, we examined the target trough concentration attainment rate in patients with FN and concomitant TAC use, which was identified as a factor influencing TEIC blood concentration in the multivariate analysis. The patients who did not receive TAC and developed FN had the lowest target trough concentration attainment rate. Therefore, these patients were grouped according to the cumulative loading dose to assess the target trough concentration attainment rates. The target trough concentration attainment rate increased in a TEIC dose-dependent manner, reaching 85% at a cumulative loading dose of 45 mg/kg. Most patients in this study were administered based on cumulative loading dose calculated according to the previous guidelines. Even under the previous dosing regimen, the finding that approximately 85% of patients administered a cumulative loading dose of 45 mg/kg achieved the target trough concentration was considered one piece of evidence supporting the necessity of the higher dosing regimen of 48–50 mg/kg recommended in the current guidelines. Among patients with a cumulative loading dose exceeding 45 mg/kg, none achieved an initial trough concentration exceeding 40 µg/mL, and no adverse events such as renal or hepatic dysfunction occurred. These findings suggest that even in patients with hematological malignancy and factors predisposing them to poor TEIC concentrations, administering a sufficient loading dose can safely increase the rate of achieving the target concentration. To the best of our knowledge, no previous study has examined patients with multiple risk factors for low TEIC concentrations, highlighting the novelty of our findings. Determining an effective and safe loading dose for these patients remains a topic for future investigations.

This study had several limitations. First, this was a retrospective study conducted at a single center. Second, because the relationship between TEIC trough concentration and clinical efficacy was not evaluated, it is unclear whether low TEIC trough concentrations directly influence the emergence of resistant bacteria or the prolongation of infection. Third, most of the periods under review were based on the 2018 Antimicrobial TDM Clinical Practice Guidelines for dosage design, and the loading doses were lower than those recommended in the current 2022 Antimicrobial TDM Clinical Practice Guidelines. A detailed evaluation of the clinical mechanisms and a re-evaluation based on the current guidelines will be topics for future research. Finally, renal function is the primary determinant of TEIC pharmacokinetics. Furthermore, since guidelines specify a different dosing regimen for patients with an eGFR < 60 mL/min/1.73 m², this study excluded patients with an eGFR < 60 mL/min/1.73 m². Consequently, the findings of this study are primarily applicable to patients with preserved renal function, and caution is warranted when extrapolating these results to patients with moderate to severe renal impairment.

## Conclusions

In patients with hematological malignancies, the TEIC cumulative loading dose, presence of FN, concomitant TAC use, and a history of BMT within 30 days were identified as independent factors affecting TEIC trough concentration. In particular, as the target trough concentration attainment rate was lower in patients with FN who were not taking TAC concomitantly, high-dose regimens should be actively adopted, and loading doses should be administered to patients with these factors.

## Data Availability

No datasets were generated or analysed during the current study.

## Abbreviations

AML: Acute myeloid leukemia
AUC: Area under the concentration-time curve
Alb: Albumin
BMI: Body mass index
BMT: Bone marrow transplantation
FN: Febrile neutropenia
MRCNS: Methicillin-resistant coagulase-negative Staphylococci
TAC: Tacrolimus
TEIC: Teicoplanin
eGFR: Estimated glomerular filtration rate
TDM: Therapeutic drug monitoring
